# Native Hawaiian and Other Pacific Islander Representation Among US Allopathic Medical Schools, Residency Programs, and Faculty Physicians

**DOI:** 10.1001/jamanetworkopen.2021.25051

**Published:** 2021-09-20

**Authors:** Kekoa Taparra, Curtiland Deville

**Affiliations:** 1 Department of Radiation Oncology, Stanford University, Palo Alto, California; 2 Department of Radiation Oncology and Molecular Radiation Sciences, Johns Hopkins University, Baltimore, Maryland

## Abstract

This cross-sectional study analyzes the representation of Native Hawaiian and Other Pacific Islander allopathic medical students, residents, and physicians in the US physician workforce.

## Introduction

Native Hawaiian and Other Pacific Islander individuals are 1 of 5 official US racial categories, as designated by the US Census Bureau and Office of Management and Budget, and remain underacknowledged in health care research.^1^ Native Hawaiian and Other Pacific Islander individuals (who are of Melanesian, Micronesian, and Polynesian descent) are not synonymous with Asian individuals.^2^ They experience disproportionate health disparities yet are the least represented racial group in the medical professions.^1,3,4^ Native Hawaiian individuals compose 20% of the population of Hawaiʻi but less than 4% of the physician workforce.^3^ National physician workforce diversity research inappropriately aggregates or excludes Native Hawaiian and Other Pacific Islander individuals.^1,5^ Here, we analyze the representative quotients (RQs) of allopathic medical students, residents, and physicians who identify as Native Hawaiian and Other Pacific Islander.

## Methods

The Gundersen Health System institutional review board approved this cross-sectional study, which was conducted and reported according to Strengthening the Reporting of Observational Studies in Epidemiology (STROBE) reporting guideline. Publicly available medical student matriculant, resident, and academic faculty data between January 1, 2000, and December 31, 2020, were obtained by the Association of American Medical Colleges (AAMC); informed consent was waived by Gundersen Health System because the data were deidentified and publicly available.^4,6^ Participants self-identified as Native Hawaiian and Other Pacific Islander via the AAMC National Graduate Medical Education Census^6^ and US Census Bureau/Office of Management and Budget^2^ criteria.

As reported previously,^5^ an RQ is the proportion of a subgroup compared with the US population: an RQ of 1 denotes equal representation; greater than 1, overrepresentation; and less than 1, underrepresentation. Representative quotient denominators reflected 2000-2020 US census data.^2^ Linear regression was used to assess time vs RQ of Native Hawaiian and Other Pacific Islander individuals. Medians, ranges, RQ slope estimates, *P* values, and 95% CIs were calculated. All tests were 2 tailed with Bonferroni correction for multiple testing (Q values in the Table). Significance was set at *P* = .05. All analyses were conducted with R version 4.0.3 in RStudio (R Project for Statistical Computing).

**Table.** Linear Regression Analysis of Representative Quotient Slope Estimates for Native Hawaiian and Other Pacific Islander Medical Students (2002-2020), Residents (2002-2020), and Academic Faculty (2000-2018)

Characteristic	RQ slope estimate (95% CI)	*P* value	Q value^a^
Medical students			
Alone	−6.3 (−12 to −0.44)	.05	0.15
In combination	10 (0.15 to 20)	.06	0.19
Alone or in combination with 1 or more races	−7.4 (−22 to 7.5)	.35	>0.99
Residents			
Anesthesia	−5.8 (−7.6 to −3.9)	<.001	<0.001
Dermatology	−3.7 (−7.0 to −0.48)	.04	0.77
Emergency medicine	−5.2 (−7.9 to −2.5)	.002	0.04
Family medicine	−1.5 (−2.3 to −0.61)	.004	0.09
Internal medicine	−2.8 (−4.6 to −1.1)	.007	0.13
Neurosurgery	−3.8 (−5.8 to −1.9)	.002	0.03
Neurology	−4.3 (−6.7 to −1.9)	.003	0.07
Obstetrics/gynecology	−5.0 (−7.1 to −2.9)	<.001	0.01
Ophthalmology	−2.9 (−4.3 to −1.6)	<.001	0.01
Orthopedic surgery	−6.4 (−8.5 to −4.2)	<.001	<0.001
Otolaryngology	−6.1 (−11 to −1.3)	.02	0.47
Pathology	−4.5 (−6.0 to −3.0)	<.001	<0.001
Pediatrics	−3.4 (−4.8 to −2.0)	<.001	0.01
Physical medicine and rehabilitation	−2.5 (−3.6 to −1.4)	<.001	0.01
Plastic surgery	−5.2 (−7.7 to −2.7)	<.001	0.02
Psychiatry	−7.1 (−8.7 to −5.4)	<.001	<0.001
Radiation oncology	−2.9 (−4.5 to −1.2)	.004	0.08
Radiology	−7.4 (−11 to −3.9)	<.001	0.01
General surgery	−9.1 (−13 to −4.7)	<.001	0.02
Urology	−5.4 (−11 to 0.39)	.09	>0.99
Academic faculty			
Anesthesia	−15 (−39 to 7.9)	.22	>0.99
Dermatology	4.9 (1.4 to 8.4)	.02	0.28
Emergency medicine	16 (−5.0 to 36)	.16	>0.99
Family medicine	−3.5 (−23 to 16)	.72	>0.99
Internal medicine	14 (7.2 to 20)	<.001	0.02
Neurosurgery	4.6 (−1.9 to 11)	.19	>0.99
Neurology	13 (5.5 to 21)	.005	0.09
Obstetrics/gynecology	−1.3 (−9.3 to 6.6)	.75	>0.99
Ophthalmology	0.67 (−16 to 17)	.94	>0.99
Orthopedic surgery	−12 (−41 to 18)	.46	>0.99
Otolaryngology	0.54 (−12 to 13)	.93	>0.99
Pathology	−12 (−25 to 0.61)	.08	>0.99
Pediatrics	10 (0.73 to 19)	.05	0.95
Physical medicine and rehabilitation	−3.0 (−6.8 to 0.79)	.14	>0.99
Plastic surgery	NA^b^	NA^b^	NA^b^
Psychiatry	−9.5 (−26 to 7.2)	.28	>0.99
Radiation oncology	−1.6 (−4.2 to 1.0)	.24	>0.99
Radiology	NA^b^	NA^b^	NA^b^
General surgery	13 (−1.7 to 28)	.11	>0.99
Urology	−3.0 (−9.4 to 3.4)	.38	>0.99

Abbreviations: NA, not available; RQ, representative quotient.

^a^ Bonferroni correction for multiple testing.

^b^ Not available owing to no recorded Native Hawaiian and Other Pacific Islander representation to evaluate RQ trend. Medical students are reported by Native Hawaiian and Other Pacific Islander racial categories of alone, in combination, and alone or in combination with 1 or more races. Residents and academic faculty are reported by the 20 largest specialties.

## Results

All participants in the study self-identified as Native Hawaiian and Other Pacific Islander. Between 2000 and 2020, there was overall underrepresentation of Native Hawaiian and Other Pacific Islander individuals; the numbers (RQs) for medical students, residents, and faculty physicians were 41 individuals (0.39), 82 individuals (0.37), and 85 individuals (0.40), respectively (Table; Figure, A).

**Figure.**
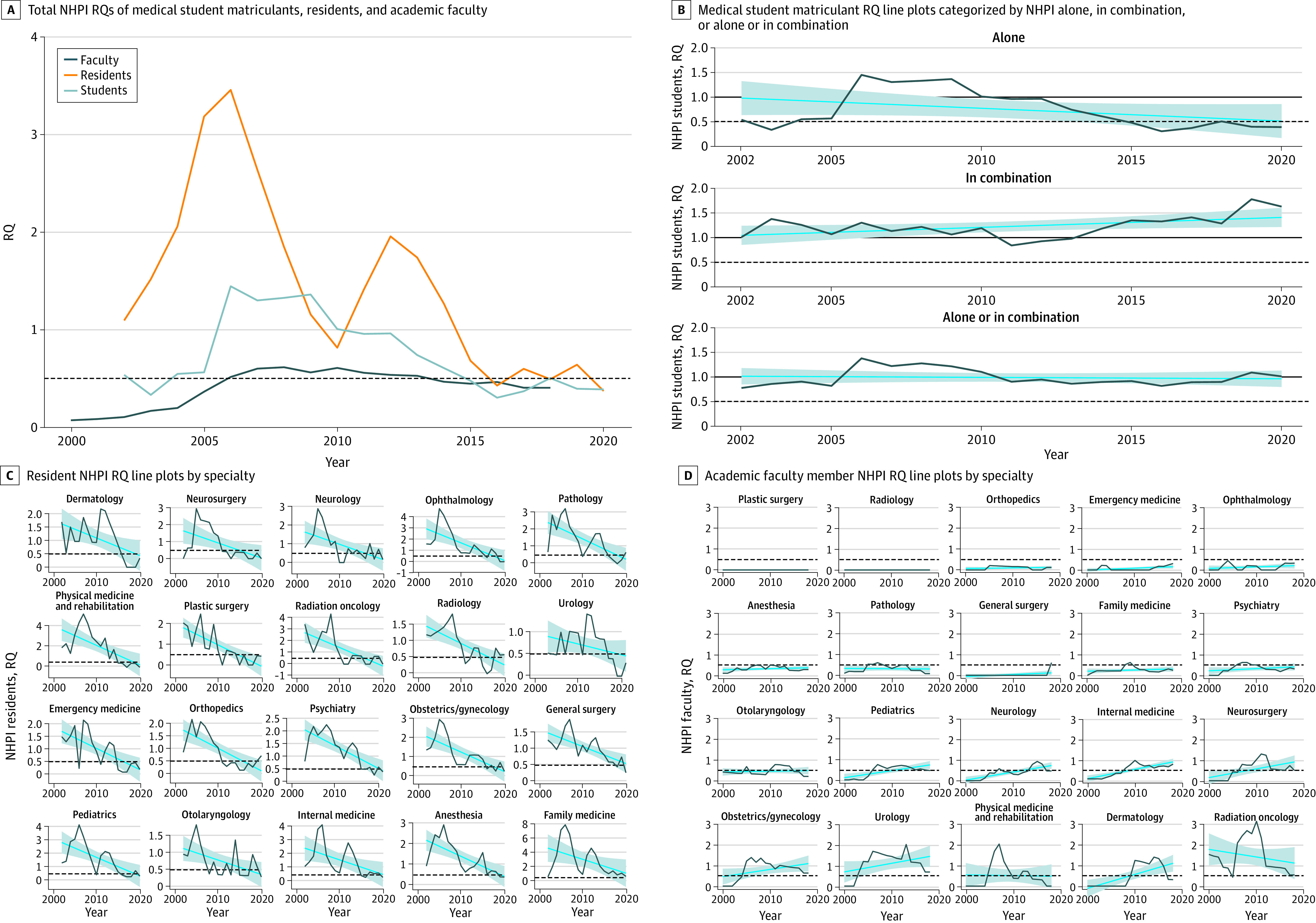
Representative Quotient (RQ) of Native Hawaiian and Other Pacific Islander (NHPI) Individuals Across Medical Specialties Specialties are arranged from least to most represented. The RQ of 1 denotes an equal proportion of Native Hawaiian and Other Pacific Islander representation compared with the US population. The dashed line represents RQ = 0.5, denoting half of the Native Hawaiian and Other Pacific Islander representation compared with the US population. In panels B-D, the blue lines represent the linear regression over time, with 95% CIs represented by the shaded area.

There were no significant changes in the number of medical student matriculants identifying as Native Hawaiian and Other Pacific Islander alone (individuals who reported Native Hawaiian and Other Pacific Islander as their only race) (RQ slope, −6.3; 95% CI, −12 to −0.44; Q = 0.15), in combination (individuals who reported Native Hawaiian and Other Pacific Islander alone or in combination with ≥1 other race) (RQ slope, 10; 95% CI, 0.15-20; Q = 0.19), and alone or in combination (RQ slope, −7.4; 95% CI, −22 to 7.5; Q>0.99) between 2002 and 2020 (Figure, B). The median annual total medical student matriculants for Native Hawaiian and Other Pacific Islander individuals alone and alone or in combination were 53 (range, 23-115) and 184 (range, 104-232), respectively. The median annual RQs for Native Hawaiian and Other Pacific Islander alone and alone or in combination were 0.56 (range, 0.30-1.44) and 0.90 (range, 0.77-1.37), respectively.

The Native Hawaiian and Other Pacific Islander resident RQ decreased across all specialties between 2002 and 2020 (Figure, C). Internal medicine, family medicine, pediatrics, and general surgery contributed approximately half of Native Hawaiian and Other Pacific Islander residents annually. Median annual Native Hawaiian and Other Pacific Islander residents across specialties was 234 (range, 81-582), with a median RQ of 1.27 (range, 0.37-3.46). After adjusting for multiple testing, most specialties had significantly decreasing RQs.

Most specialties showed a decrease to an RQ of less than or equal to 0.5 among Native Hawaiian and Other Pacific Islander faculty between 2000 and 2018 (Figure, D). The median RQ was 0.47 (range, 0.10-0.61). There were no recorded Native Hawaiian and Other Pacific Islander academic faculty in plastic surgery, radiology, or orthopedic surgery. The most represented specialties included pediatrics, internal medicine, obstetrics/gynecology, and psychiatry.

## Discussion

In this study, we detail the substantial underrepresentation of Native Hawaiian and Other Pacific Islander individuals among US medical students, residents, and faculty. Medical students identifying as Native Hawaiian and Other Pacific Islander alone vs alone or in combination had an RQ of less than 0.5 vs 1.0, respectively, underscoring the nuance of these data; more individuals who are Native Hawaiian and Other Pacific Islander self-identify as multiracial compared with individuals in other US racial groups.^2^ The number of Native Hawaiian and Other Pacific Islander residents appears to be significantly declining and unequally distributed across specialties. Their representation should be prioritized, particularly in specialty programs, through early mentorship, recruitment, and retainment. Despite being critical to mentoring indigenous trainees, Native Hawaiian and Other Pacific Islander faculty representation has not improved in the past 2 decades. This study is limited by the fact that Native Hawaiian and Other Pacific Islander residents and faculty who self-identified as multiracial in the AAMC National Graduate Medical Education Census were masked within a separate “multiracial” group and were unable to be included in this study owing to AAMC data collection methods.^1,5,6^ It would be beneficial for organizations to increase transparency and granularity when reporting data on race and ethnicity, which will facilitate more accurate tracking of Native Hawaiian and Other Pacific Islander representation without excluding those who identify as multiracial. Overall, equal representation is important, given that when Native Hawaiian and Other Pacific Islander patients are treated by racially concordant physicians, there is improvement in patient-reported satisfaction, quality, and health care outcomes.^1^ With the paucity of Native Hawaiian and Other Pacific Islander individuals in medicine, advocacy is challenging but necessary for the health of their growing communities. Future studies are warranted to elucidate systemic barriers to the Native Hawaiian and Other Pacific Islander physician workforce pipeline.
